# Complete mitochondrial genome sequence of *Parumbrosa polylobata*

**DOI:** 10.1080/23802359.2019.1627925

**Published:** 2019-07-12

**Authors:** Song Feng, Jianing Lin, Song Sun, Qing Liu

**Affiliations:** a CAS Key Laboratory of Marine Ecology and Environmental Sciences, Institute of Oceanology, Chinese Academy of Sciences, Qingdao, China;; b Laboratory for Marine Ecology and Environmental Science, Qingdao National Laboratory for Marine Science and Technology, Qingdao, China;; c Center for Ocean Mega-Science, Chinese Academy of Sciences, Qingdao, China;; d Department of Riverine Ecological Conservation and Restoration, Chinese Research Academy of Environmental Sciences, Beijing, China;; e College of Earth and Planetary Sciences, University of Chinese Academy of Sciences, Beijing, China;; f College of Environmental Science and Engineering, Yangzhou University, Yangzhou, China

**Keywords:** *Parumbrosa polylobata*, complete mitochondrial genome, *Aurelia* spp

## Abstract

The complete mitochondrial genome sequences of giant jellyfish *Parumbrosa polylobata*, a scyphozoan species inhabiting the Yellow Sea cold bottom water in China, is firstly described and analyzed in this research. It is 16,809 bp in length with a linear mitochondrial DNA. The base composition of the genome with A + T bias is 69.7%. There are 13 protein-coding genes (PCGs), 2 tRNAs, and 2 rRNAs. Another two special PCGs, *ORF360* and *ORF147* with telomeres were found at both ends. The neighbor-joining (NJ) phylogenetic tree among the related 13 species showed that *P. polylobata* is close to *Aurelia* spp.

The giant jellyfish *Parumbrosa polylobata* belongs to Cnidaria, Scyphozoa, Semaeostomeae, Ulmaridae. It was a predominant scyphozoan species in Yellow Sea, China, and is mainly distributed in the Yellow Sea cold bottom water with preference for cool temperature and higher salinity (Zhang et al. 2012).

It is imperative to study complete mitochondrial DNA of scyphozoan species to have a good knowledge of molecular phylogenetic relationships among them. However, there were only several species involved until now, such as *Aurelia* spp. (Shao et al. 2006; Hwang, Park, Won, Lee JS 2014), *Craspedacusta sowerbyi* (Zou et al. 2012), *Chrysaora quinquecirrha* (Hwang, Park, Won, Lee WJ, et al. 2014), *Nemopilema nomurai*, and *Rhopilema esculentum* (Wang and Sun 2017a, 2017b). In this study, we firstly report the complete mitochondrial genome from *P. polylobata* to obtain the basic genetic information of *P. polylobata* population in Yellow Sea, China. Medusae of *P. polylobata* were collected from the central Yellow Sea (35.5° N, 124° E), preserved with 95% ethanol and placed in a −40 °C refrigerator. Then they were stored in an aquarium in the Institute of Oceanology, Chinese Academy of Sciences. The bell tissue of medusae was used for further processing of mitochondrial DNA.

The complete mitochondrial genome of *P. polylobata* was 16,809 bp in length with a linear mitochondrial DNA (GenBank accession No. MK689180). It consists of 13 protein-coding genes (PCGs), 2 transfer RNA genes (tRNA-TCA, tRNA-TTT), 2 ribosomal RNA genes (small subunit RNA and large subunit RNA), and 2 additional PCGs, *ORF147* and *ORF 360* with telomeres at both ends. In the 13 PCGs, there are 7 PCGs starting with ATG codon (*COX1*, *COX2*, *ATP6*, *COX3*, *ND5*, *ND4L*, and *Cytb*), 3 with ATA codon (*ND2*, *ND1*, and *ND4*), 2 with ATT codon (*ATP8* and *ND6*), and 1 with ATC codon (*ND3*). All genes exhibited complete stop codons using TAA (*COX1*, *ATP6*, *COX3*, *ND2*, *ND5*, *ND3*, *ND4L*, *ND1*, and *ND4*) and TAG (*ATP8*, *ND6*, and *Cytb*) except *COX2* with T (AA) codon. Moreover, the slight anti-G bias was found in the 3rd position of PCGs (12.7%). The mitochondrial genome base composition for 13 PCGs was 31.9% for A, 37.8% for T, 16.1% for G, and 14.2% for C. The A + T base composition (69.7%) was higher than G + C (30.3%), suggesting that the *P. polylobata* has significant low G + C ratio in the mitochondrial genome similar to *Aurelia* spp. (Shao et al. 2006). The neighbor-joining (NJ) phylogenetic tree among 13 species was generated based on the complete mitochondrial genome from NCBI (Figure 1). The results showed that *P. polylobata* is close to *Aurelia* spp. (GenBank No. DQ787873.1, HQ694729.1, LC005413.1 and LC005414.1).

**Figure 1.**
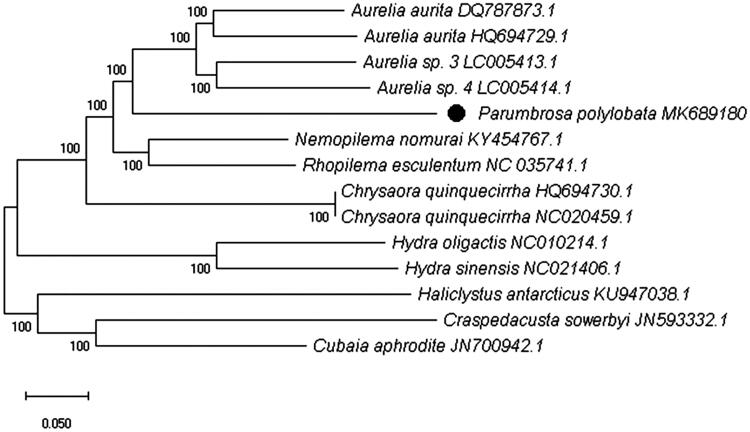
Phylogenetic relationship revealed by NJ tree.
